# Anesthesia management of an aged patient with giant abdominal tumor and large hiatal hernia: A case report and literature review

**DOI:** 10.3389/fsurg.2022.921887

**Published:** 2022-10-25

**Authors:** Bingbing Xiang, Mingliang Yi, Hong Yin, Rui Chen, Feng Yuan

**Affiliations:** Department of Anesthesiology, Chengdu Fifth People's Hospital (The Second Clinical Medical College, Geriatric Diseases Institute of Chengdu/Cancer Prevention and Treatment Institute of Chengdu, Affiliated Fifth People's Hospital of Chengdu University of Traditional Chinese Medicine), Chengdu, China

**Keywords:** aggressive fibromatosis, hiatal hernia, anesthesia management, arrhythmia, case report

## Abstract

**Introduction:**

A giant abdominal tumor with a large hiatal hernia remains a rare disease with few studies regarding its implications in anesthesia. A large hiatal hernia may compress the heart and cause arrhythmia and even cardiac arrest, which greatly increases the risks and challenges of anesthesia management.

**Case description:**

We present a case in which a patient with a giant abdominal desmoid tumor and large hiatal hernia experienced a critical situation during anesthesia and surgery.

**Conclusions:**

It is a great challenge for anesthesiologists to manage a patient's respiratory system and circulation. Careful perioperative management and optimized multidisciplinary teams are the key factors in the successful management of this rare condition. In addition, awake endotracheal intubation, ventilation preserving spontaneous breathing and target-directed fluid therapy play an essential role in anesthesia management.

## Introduction

**Figure 1:**
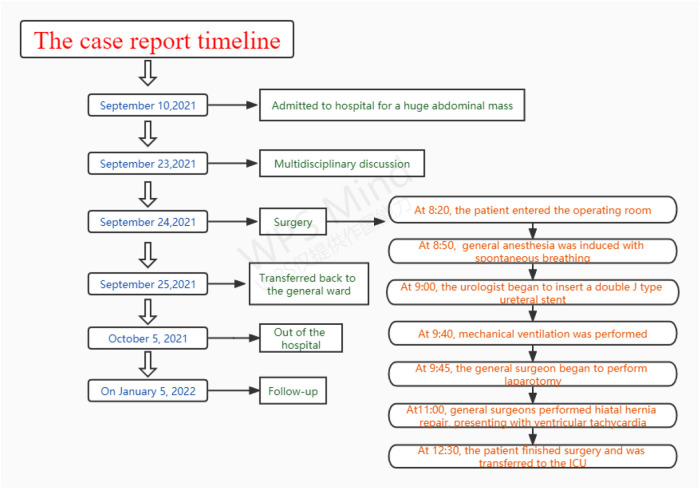
The case report timeline with relevant data from the episode of care.

Hiatal hernia is not a rare complication, but it can be a serious complication of giant abdominal tumors caused by increased intra-abdominal pressure (1). A large hiatal hernia can increase intrathoracic pressure and compress the heart and even cause arrhythmia and cardiac arrest, which greatly increases the risks of anesthesia and the challenges of perioperative management (2–5). We present a case in which a patient with a giant abdominal tumor and large hiatal hernia experienced a critical situation during anesthesia and surgery (Figure 1).

Written informed consent was obtained from the patient, and this manuscript adheres to the CARE reporting guidelines.

## Case description

**Figure 2:**
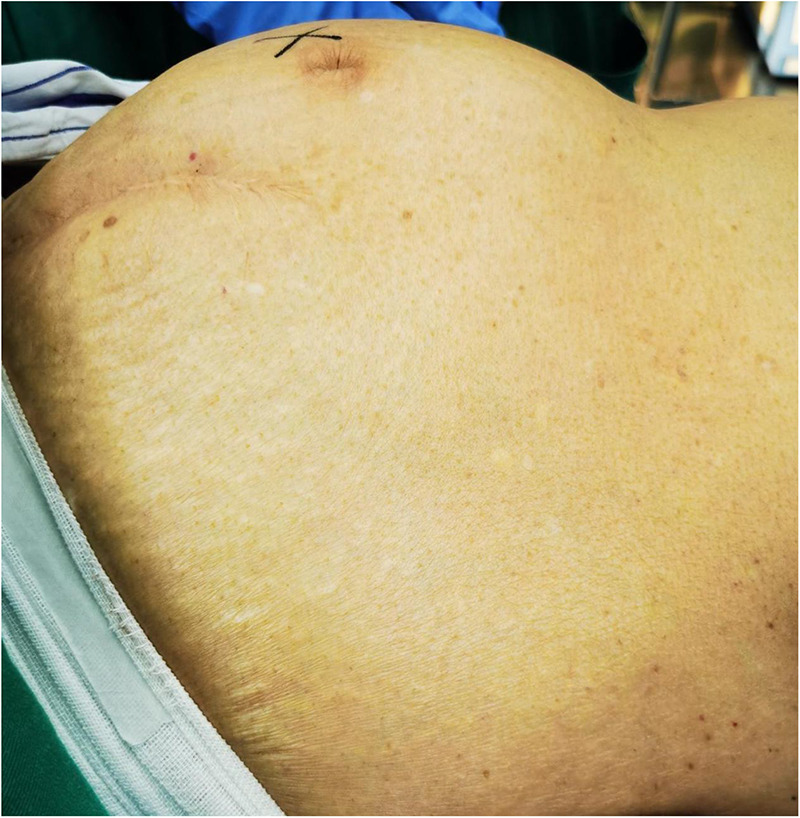
Physical examination showed obvious periumbilical eminence and palpable abdominal mass measured about 30 cm × 30 cm × 30 cm.

**Figure 3:**
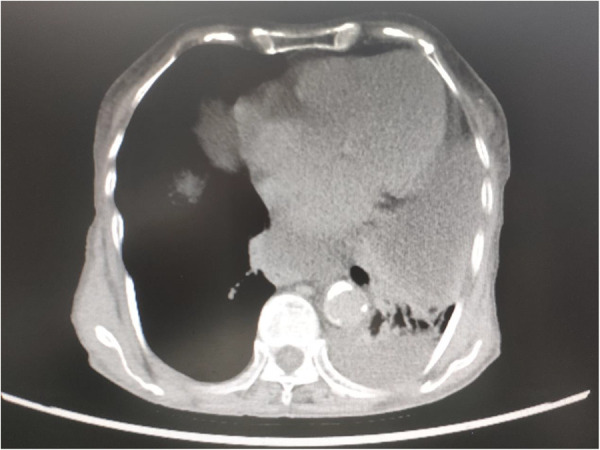
CECT of the chest revealed a large hiatal hernia within the intrathoracic stomach located just behind the heart, and part of the ascites herniated into the thoracic cavity.

An 82-year-old female (weight, 41.5 kg; height, 155 cm) was admitted for a large abdominal mass (Figure 2). The patient had a history of hypertension and valvular heart disease. Contrast-enhanced computed tomography (CECT) scans of the abdomen revealed a giant tumor (15.5 cm × 18.1 cm × 26.5 cm) located in the abdominal and pelvic cavities with moderate ascites. CECT of the chest revealed a large hiatal hernia within the intrathoracic stomach located just behind the heart and part of the ascites herniated into the thoracic cavity (Figure 3). Doppler echocardiography was also performed and revealed an enlarged left atrium, severe tricuspid regurgitation, moderate pulmonary hypertension, moderate mitral regurgitation, and decreased left ventricular diastolic function. In addition, pulmonary function tests demonstrated mild obstructive ventilatory dysfunction and a severe reduction in ventilatory reserve rate (56%). The laboratory examinations revealed that the NT-proBNP level was 5475 cpg/ml.

According to the recommendation of the multidisciplinary team (MDT), the abdominal tumor was first excised under general anesthesia with tracheal intubation. Since the neoplasms may have invaded the ureter, the urologist intended to insert a double *J*-type ureteral stent prior to surgery.

After the patient entered the operating room at 08:20, noninvasive blood pressure (BP), electrocardiogram (ECG) and saturation of pulse oxygen (SpO2) were monitored. Her heart rate was 104 beats/min, her blood pressure was 150/81 mmHg, and her oxygen saturation was 89% under air inhalation. Before general anesthesia, radial artery catheterization and deep vein catheterization were performed under local anesthesia for continuous invasive blood pressure and cardiac output (CO) monitoring. At this point, her CO was 4.65 L/min, systemic vascular resistance (SVR) was 1,326 BSA, stroke volume (SV) was 57 ml, and stroke volume variation (SVV) was 13.9%.

Anesthesia induction was performed with continuous remifentanil pumping at 0.1 µg/kg/min and dexmedetomidine pumping at 0.2 µg/kg/h at 08:50. The mucosa of the oral cavity, root of the tongue and throat were surface anesthetized with a 2% lidocaine nebulizer. Then, 2 ml of 2% lidocaine was extracted with a 5 ml syringe for thyrocricoid puncture, and endotracheal surface anesthesia was fully performed as the patient coughed. Minutes later, endotracheal intubation was performed using a fiberoptic bronchoscope while the patient was under sedation and still breathing spontaneously. Her breathing rate was 15 times/min, end-tidal carbon dioxide partial pressure (ETCO2) was 34 mmHg, SpO2 was 100%, BP was 127/47 mmHg, HR was 98 beats/min, CO was 4.98 L/min, SVR was 1,502 BSA, SV was 55 ml, and SVV was 12.9%. Subsequently, the patient was given continuous inhalation of 2% sevoflurane, and the urologist began to insert a double *J*-type ureteral stent.

At 9:35, the patient's breathing rate gradually decreased to approximately 10 times/min, with ETCO2 at 46 mmHg. We had to manually pinch the breathing balloon for ventilatory support. After her breathing rate decreased to 7 times/min at 9:40, mechanical ventilation was performed with an intravenous injection of 10 ug sufentanil. Due to the high airway pressure of 37 mmHg, 30 mg rocuronium was added intravenously. The patient's airway pressure was reduced to normal, with SpO2 being 100% and ETCO2 being 33 mmHg. However, her blood pressure dropped suddenly to 59/21 mmHg; her heart rate rose rapidly to 132 beats/min; and her CO was 2.34 L/min, SVR was 2,595 BSA, SV was 20 ml, and SVV was 15.9%. Immediately, 6 mg ephedrine was injected intravenously to raise blood pressure, and the general surgeons were requested to perform laparotomy to relieve intra-abdominal pressure. At 9:45, hemodynamics gradually stabilized after laparotomy and decompression. At that time, the patient's BP was 133/46 mmHg, HR was 81 beats/min, CO was 3.11 L/min, SVR was 2,875 BSA, SV was 33 ml, and SVV was 7.07%. When the abdominal mass was removed at 10:40, the patient's BP was 95/27 mmHg, HR was 77 beats/min, CO was 2.51 L/min, SVR was 2,222 BSA, SV was 33 ml, and SVV was 15.5%. Considering the patient's lack of volume due to SVV over 13%, fluid infusion of 500 ml was accelerated. Subsequently, the patient's hemodynamics stabilized, and SVV decreased to 7%. At 11:00, frequent premature ventricular followed by ventricular tachycardia were observed during hiatal hernia repair by general surgeons. We immediately ordered the surgeon to suspend the procedure, and the patient's heart rhythm returned to normal.

After surgery, the patient was transferred to the intensive care unit (ICU) with ventilation at 12:30, and her SpO2 was 100%, ETCO2 was 29 mmHg, BP was 101/30 mmHg, HR was 64 beats/min, CO was 2.90 L/min, SVR was 1,915 BSA, SV was 46 ml, and SVV was 6.59%. The endotracheal tube was removed in the ICU on the second day after surgery, and the patient was transferred back to the general ward. The patient fully recovered and was discharged home on the tenth day after surgery.

## Follow-up

After being followed up for 3 months, the patient was free of abdominal discomfort, sweating, acid reflux, neurological complications or symptoms of heart failure. Meanwhile, there were no signs of recurrence through CT evaluation.

## Discussion and conclusions

**Figure 4:**
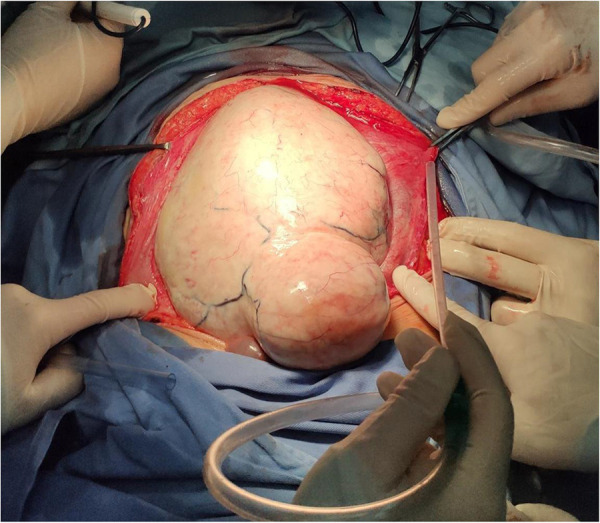
A single, giant tumor with smooth surface was observed after laparotomy, which eventually diagnosed as aggressive fibromatosis, characterized by hard and fixed masses with a long history.

The patient was diagnosed with a giant abdominal tumor with large hiatal hernia and suffered from hypertension and valvular heart disease. In addition, postoperative histopathology of the tumor revealed a spindle cell tumor, and the patient was eventually diagnosed with aggressive fibromatosis (Figure 4). Aggressive fibromatosis, also known as desmoid tumor or desmoid type fibromatosis, was first described by MacFarlane in 1,832 (6). It is a rare tumor of fibroblast origin; the tumor is benign pathologically, with no distant metastasis but local invasive growth (7, 8). The clinical symptoms of patients are usually characterized by hard and fixed masses with a long history, and some are accompanied by pain (9). The tumor can occur anywhere in the body, and the most common sites are the proximal extremities and abdomen (8, 9).

The patient developed a large hiatal hernia due to the abdominal mass that increased intra-abdominal pressure. CECT of the chest demonstrated that the stomach and part of the ascites had herniated into the thoracic cavity from the esophageal hiatus. It has been widely reported that gastrothorax can increase intrathoracic pressure and compress the heart and can even cause cardiac arrest (2, 3, 5, 10). The increased BNP level on admission in this case might indicate diastolic dysfunction due to cardiac compression (11). Once positive pressure mechanical ventilation is applied to the patient, the dramatically boosted intrathoracic pressure will crush the heart violently and cause a sharp drop in blood pressure. Therefore, auxiliary ventilation preserving the patient's spontaneous breathing was performed during the induction of anesthesia. When the patient is awake and breathing spontaneously, the diaphragm has a strong contractile force, which can prevent further tissue herniation and excessive pressure in the chest cavity (12, 13). Therefore, we adopted the awake endotracheal intubation technique and avoided the use of muscle relaxants. However, due to prolonged sedation and anesthesia, the patient's breathing rate became progressively slower, which was something we did not foresee. After 50 min, positive pressure mechanical ventilation was eventually needed, and the muscle relaxant was administered intravenously. At this point, the patient's hemodynamics deteriorated suddenly and then were rapidly resuscitated, followed by laparotomy and decompression by the general surgeons.

A multidisciplinary team is very important for assessing perioperative risks and conducting individualized treatment protocols. The large mass compressed the gastrointestinal tract, resulting in delayed gastric emptying. Therefore, the patient was treated as having a full stomach to prevent aspiration of gastric contents during anesthesia induction. Awake endotracheal intubation can effectively avoid this situation. For severe valvular heart disease and hypertensive heart disease, the total volume and speed of fluid should be controlled during the perioperative period to avoid heart failure. Therefore, invasive arterial blood pressure and cardiac output were monitored dynamically during surgery. Based on continuous monitoring of CO and SVV, target-directed fluid therapy was adopted to maintain fluid balance and reduce cardiac volume load. Furthermore, the circulation and blood pressure plummeted rapidly when the tumor was removed due to a sharp decrease in the compression of the abdominal aorta and inferior vena cava. The patient was closely monitored at this time, and over time, fluids were replenished. Although rarely reported, patients with paraesophageal hernia may experience arrhythmia, including sinus tachycardia, atrial flutter, atrial fibrillation, supraventricular extrasystole and ventricular tachycardia (2, 4, 14–21). In this case, frequent premature ventricular followed by ventricular tachycardia were observed during the operation, which were closely monitored. Gnanenthiran et al (14). also reported a novel case of ventricular tachycardia caused by posterior cardiac compression from a large hiatal hernia. The literature written in English published from 1989 to 2022 was reviewed, and approximately 10 cases of arrhythmias caused by hiatal hernia have been reported (Table 1) (2, 4, 14–21).

**Table 1:** Clinical features of arrhythmia caused by hiatal hernia from published case reports.

Ref.	Year	Age	Sex	ECG	ECHO	CT or x-ray	Clinical features
Gleadle J et al. (15)	1989	65	F	Sinus tachycardia	Hiatal hernia compressing and displacing the heart	Very large hiatal hernia	Vomiting
Schilling RJ et al. (16)	1998	72	M	Atrial flutter with 2:1 AV block	Normal	Large mediastinal mass	Heart palpitations
Tursi A et al. (17)	2001	75	F	Supraventricular extrasystole, atypical right bundle branch block pattern	No information	Giant gastric hiatal hernia compressing LA	Weakness, dysphagia, heartburn
Gürgün C et al. (18)	2002	76	F	Atrial fibrillation	Normal	Cardiomegaly, a dome shaped air level overlapping cardiac silhouette	Dyspnea
Duygu H et al. (19)	2008	79	F	Atrial fibrillation	TR, MR, high PAP	Hiatal hernia	Chest pain
Patel A et al. (4)	2014	80	F	Atrial flutter	High PAP	Large hiatal hernia	Failure to thrive and weakness
Cristian DA et al. (20)	2015	77	F	Atrial fibrillation	Mass compressing LA, MR	Large shadow overlapping the heart	Dyspnea
Schumer W (21)	2017	73	M	Left bundle branch block	No information	Large hiatal hernia	Dyspnea, chest pain
Gnanenthiran SR et al. (14)	2018	78	M	Ventricular tachycardia	Mass compressing LA, dyskinesis of LV	No information	Syncope
KrawiecK. et al. (2)	2021	51	M	Sinus tachycardia, incomplete right bundle branch block	No information	Giant hiatal hernia compressing and displacing the heart	Abdominal pain

F, female; M, male; LA, left atrium; TR, tricuspid regurgitation; MR, mitral regurgitation; PAP, pulmonary arterial pressure; LV, left ventricle.

In summary, giant abdominal desmoid tumors with large hiatal hernias remain a rare disease with few studies in the literature regarding its implications in anesthesia. Early resection of desmoid tumors and repair of hiatal hernias is an optimal treatment. It is a great challenge to anesthesiologists to manage the patient's respiratory system and circulation. Careful perioperative management and optimized multidisciplinary teams are the key factors in the successful management of this rare condition. In addition, sufficient preparation, rigorous monitoring, awake endotracheal intubation, ventilation preserving spontaneous breathing and target-directed fluid therapy play an essential role in anesthesia management.

## Data Availability

The raw data supporting the conclusions of this article will be made available by the authors, without undue reservation.
